# Three Draft Genome Sequences of White Spot Syndrome Virus from India

**DOI:** 10.1128/MRA.00579-21

**Published:** 2021-08-26

**Authors:** Rajendran Kooloth Valappil, Deepika Anand, Amod Kulkarni, Manabesh Mahapatra, Sanath H. Kumar, Megha K. Bedekar, Riji John Kollanoor, Rosalind George Mulloorpeedikayil, Mansoor Mohamed Mohideenpitchai, Selvamageswaran Muthumariappan, Sivasankar Panchavarnam, Kaviarasu Devaraj, David Bass, Ronny van Aerle

**Affiliations:** a Aquatic Environment and Health Management Division, Indian Council of Agricultural Research-Central Institute of Fisheries Education, Mumbai, Maharashtra, India; b Department of Fish Pathology and Health Management, Fisheries College and Research Institute, Tamil Nadu Dr. J. Jayalalithaa Fisheries University, Thoothukudi, Tamil Nadu, India; c International Centre of Excellence for Aquatic Animal Health, Centre for Environment, Fisheries, and Aquaculture Science, Weymouth, Dorset, United Kingdom; KU Leuven

## Abstract

White spot syndrome virus (WSSV) is a pathogen causing significant economic losses to shrimp aquaculture worldwide. Previously, five genome sequences of the virus from farmed shrimp (Penaeus vannamei and Penaeus monodon) in India were reported, all originating from farms located on the east coast of the country. Here, we report three new and distinct WSSV genome sequences, two from shrimp (*P. vannamei*) farmed on the west coast of India and the third from the east coast.

## ANNOUNCEMENT

White spot syndrome virus (WSSV) is a virulent pathogen infecting farmed shrimp that causes significant economic losses worldwide and has emerged as one of the most prevalent and widespread viruses of crustaceans (1).

WSSV-infected Pacific white shrimp (*Penaeus* [*Litopenaeus*] *vannamei*) were collected from shrimp farms in Maharashtra (west coast of India) in October 2016 and January 2019 (CWG3 and PG1, respectively) and from a shrimp farm in Tamil Nadu (east coast of India) in November 2018 (DBA1182). DNA was extracted from shrimp gut tissues using a cetyltrimethylammonium bromide (CTAB)-EDTA DNA extraction protocol (2). Sequence libraries for samples CWG3 and PG1 were prepared using the NextSeq series midoutput kit (Illumina, San Diego, CA) and sequenced on an Illumina NextSeq 500 sequencer (2 × 150 bp). A sequence library for DBA1182 was prepared using the NEBNext Ultra II DNA library preparation kit and sequenced on an Illumina HiSeq sequencer (2 × 150 bp). Adapter sequences and low-quality bases were removed using fastp v0.20.0 (3). For each sample, sequences were normalized using BBNorm, part of BBTools v38.03 (4) (parameters used: ecc=t, bits=16, prefilter), prior to *de novo* assembly with SPAdes v3.13.1 (5) using the only-assembler flag and k-mers of 21, 33, 55, 77, 99, and 127. Assembled contigs were compared to a reference WSSV genome sequence (WSSV-CN [GenBank accession number AF332093.3]) using BLASTN v2.9.0+ (6) to identify contigs representing WSSV. Sequencing statistics, including the number of contigs generated, genome length, GC content, and genome coverage for each WSSV genome, are summarized in Table 1. The genome sequences of two WSSV strains (WSSV-IN-LS and WSSV-IN-NS) previously reported from India (7) could not be found in the public databases and therefore were generated by processing the sequences downloaded from the NCBI Sequence Read Archive (SRA) (SRA accession numbers SRR3233836 and SRR3233837, respectively) using the methods described above. The WSSV-IN-LS sequence consists of 12 contigs with a total length of 299,240 bp (GC content of 40.97%, with 301× coverage), whereas WSSV-IN-NS has 3 contigs totaling 284,022 bp (GC content of 41.08%, with 1,119× coverage). A core genome sequence alignment containing parts of the genome present in all selected genome sequences was generated with Parsnp v1.2 (8) using the WSSV reference genome and a selection of other WSSV genomes. A Bayesian phylogenetic tree was constructed based on the variable positions of only the core genome alignments using MrBayes v3.2.6 (9) on the CIPRES server (10). Maximum likelihood bootstrap values were calculated in RAxML BlackBox (11), also on the CIPRES server.

**TABLE 1:** Summary of sequencing statistics for each WSSV genome

WSSV isolate	Origin	No. of Illumina read pairs generated	Total no. of assembled contigs	No. of WSSV contigs	Genome length (nucleotides)	Genome coverage (×)	GC content (%)	GenBank accession no.	SRA accession no.
WSSV-IN-CWG3	Maharashtra (west coast of India)	15,466,612	34,011	2	281,060	1,596	41.05	MW248108	SRR12970171
WSSV-IN-PG1	Maharashtra (west coast of India)	8,212,139	36,748	1	285,601	286	41.10	MW248106	SRR12970170
WSSV-IN-DBA1182	Tamil Nadu (east coast of India)	17,189,702	142,544	1	281,813	3,363	41.08	MW248107	SRR12970169

**FIG 1:**
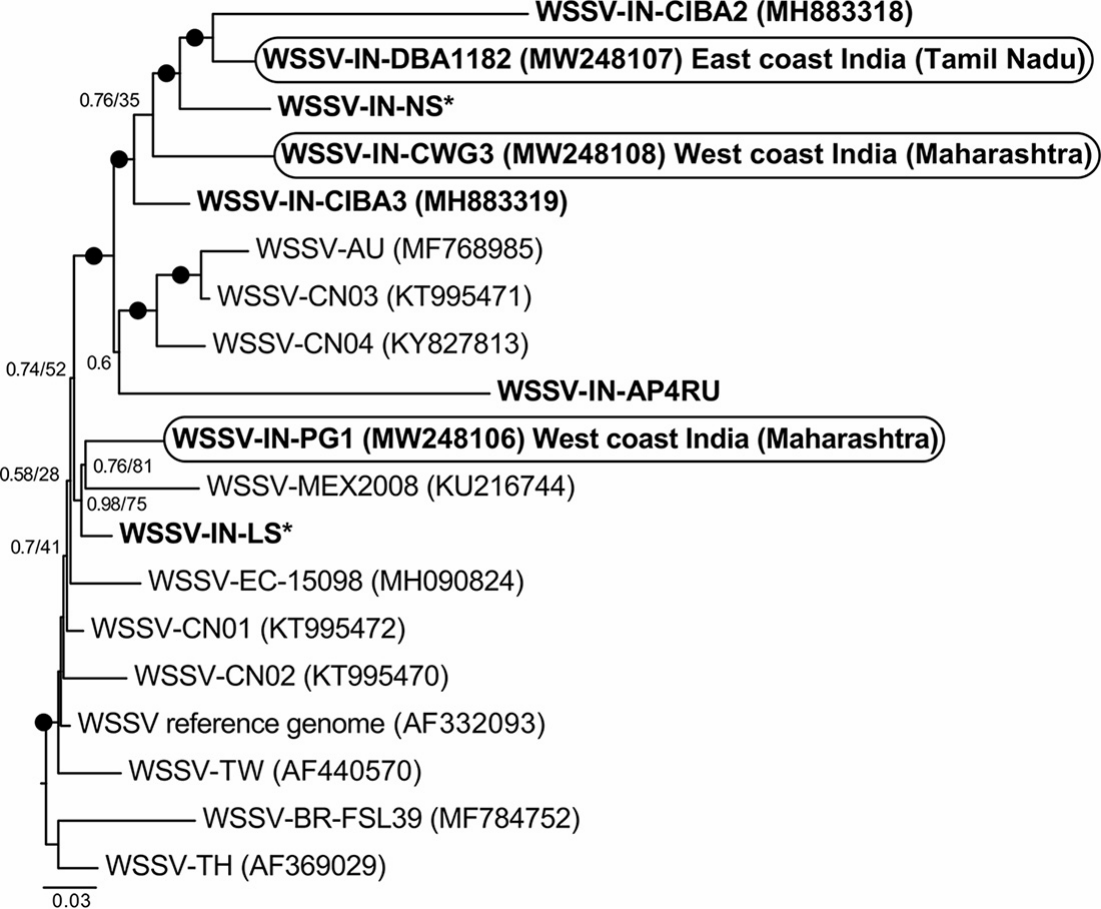
Bayesian phylogeny of all 914 variable (single-nucleotide polymorphism [SNP]) positions in the core genome alignments (total alignment length, 225,757 bp) of 19 WSSV strains, including 3 new genomes sequenced for this study (WSSV-IN-DBA1182, WSSV-IN-CWG3, and WSSV-IN-PG1) (in rounded rectangles). Bayesian posterior probability (*x*) and maximum likelihood bootstrap (*y*) values are shown at the nodes (*x*/*y*); black circles show joint bipartition support of >0.95/>95%. Branch labels indicate the country of origin (IN, India; CN, China; AU, Australia; MEX, Mexico; EC, Ecuador; TW, Taiwan; BR, Brazil; TH, Thailand). All available genome sequences from India are shown in bold. GenBank accession numbers are provided in parentheses where available. *, the assemblies of WSSV-IN-LS and WSSV-IN-NS were generated during the course of this study and have been uploaded to figshare (https://doi.org/10.6084/m9.figshare.14252951 and https://doi.org/10.6084/m9.figshare.14252966, respectively).

Phylogenetic analyses of the three new Indian WSSV genomes (Fig. 1) showed that two of them (WSSV-IN-DBA1182 and WSSV-IN-CWG3) branched robustly with three other genotypes from India. The third genome (WSSV-IN-PG1) branched with a WSSV genome (WSSV-IN-LS) that we assembled from data generated in a previous study (7) and the genome of a Mexican strain obtained from GenBank (accession number KU216744). Two of the new strains sequenced in the present study (WSSV-IN-CWG3 and WSSV-IN-PG1) were from shrimp farmed on the west coast of India, whereas all other strains reported from India were from the east coast.

### Data availability.

This whole-genome shotgun project was deposited in DDBJ/ENA/GenBank under BioProject accession number PRJNA674024. The raw reads were deposited in the SRA under accession numbers SRR12970169, SRR12970170, and SRR12970171. The assembled genome sequences for WSSV-IN-CWG3, WSSV-IN-PG1, and WSSV-IN-DBA1182 were deposited in GenBank under accession numbers MW248108, MW248106, and MW248107, respectively. Genome assemblies for WSSV-IN-LS and WSSV-IN-NS are available from figshare (https://doi.org/10.6084/m9.figshare.14252951 and https://doi.org/10.6084/m9.figshare.14252966, respectively).
